# The Glycoprotein (GP)Ib-IX-V Complex on Platelets: GPIbα Protein Expression Is Reduced in HeartMate 3 Patients with Bleeding Complications within the First 3 Months

**DOI:** 10.3390/ijms24065639

**Published:** 2023-03-15

**Authors:** Kristin Klaeske, Anneke Brade, Sandra Eifert, Khalil Jawad, Diyar Saeed, Josephina Haunschild, Franz Sieg, Michael A. Borger, Maja-Theresa Dieterlen

**Affiliations:** Department of Cardiac Surgery, Heart Center Leipzig, University of Leipzig, 04289 Leipzig, Germany; kristin.klaeske@helios-gesundheit.de (K.K.); michael.borger@helios-gesundheit.de (M.A.B.)

**Keywords:** heart failure, ventricular assist device, mechanical circulatory support, non-surgical bleeding, platelet glycoprotein

## Abstract

Non-surgical bleeding (NSB) remains the most critical complication in patients under left ventricular assist device (LVAD) support. It is well known that blood exposed to high shear stress results in platelet dysfunction. Compared to patients without NSB, decreased surface expression of platelet receptor GPIbα was observed in LVAD patients with NSB. In this study, we aimed to compare the expression level of glycoprotein (GP)Ib-IX-V platelet receptor complex in HeartMate 3 (HM 3) patients with and without bleeding complications to investigate the alterations of the platelet transcriptomic profile on platelet damage and increased bleeding risk. Blood samples were obtained from HM 3 patients with NSB (bleeder group, *n* = 27) and without NSB (non-bleeder group, *n* = 55). The bleeder group was further divided into patients with early NSB (bleeder ≤ 3 mo, *n* = 19) and patients with late NSB (bleeder > 3 mo, *n* = 8). The mRNA and protein expression of GPIbα, GPIX and GPV were quantified for each patient. Non-bleeder, bleeder ≤ 3 mo and bleeder > 3 mo were comparable regarding the mRNA expression of GPIbα, GPIX and GPV (*p* > 0.05). The protein analysis revealed a significantly reduced expression level of the main receptor subunit GPIbα in bleeders ≤ 3 mo (*p* = 0.04). We suggest that the observed reduction of platelet receptor GPIbα protein expression in patients who experienced their first bleeding event within 3 months after LVAD implantation may influence platelet physiology. The alterations of functional GPIbα potentially reduce the platelet adhesion capacities, which may lead to an impaired hemostatic process and the elevated propensity of bleeding in HM 3 patients.

## 1. Introduction

Left ventricular assist device (LVAD) implantation is an accepted therapeutic option for end-stage heart failure (HF) patients. Despite the benefits, postoperative non-surgical bleeding (NSB) and thromboembolic events remain the most critical complications in patients under LVAD support [1,2,3]. Five-year outcomes in patients with the new generation HeartMate 3 (HM 3) fully magnetically levitated centrifugal-flow pump showed an incidence of NSB in 46% of patients, mostly occurring in the first months of implantation [4,5].

Previous reports assumed that the permanent exposure of blood components to LVAD-induced non-physiological shear stress might result in an impaired coagulation system [2]. Thus, platelet dysfunction was suggested as one possible cause of major bleeding in patients with LVAD [2,3,6]. The surface of platelets contains a number of glycoproteins involved in hemostasis and thrombus formation [6]. Glycoprotein (GP)Ib-IX-V is one of the most abundant receptor complexes and is exclusively expressed on the surface of resting platelets. This complex is involved in the process of platelet adhesion, which is the initial step in response to vascular injury. The GPIb-IX-V complex contains four different subunits of type I transmembrane proteins: GPIbα, GPIbβ, GPIX and GPV [7]. GPIbα is the main subunit in this receptor complex and includes the binding domain for multimeric plasma glycoprotein von Willebrand factor (vWF) that initiates primary hemostasis [8]. LVAD-induced shear stress not only results in platelet activation and hemolysis but also triggers the shedding of platelet receptors [9]. Previous studies showed that non-physiological shear stress contributes to both in vitro and in vivo shedding of different important platelet receptor glycoproteins contribute to platelet dysfunction [10,11,12,13,14]. Further, longer exposure to high shear stress induces platelet degranulation resulting in the downregulation of primary platelet adhesion receptors and limited platelet activation [15]. Especially, platelet GPIbα surface expression is reduced through receptor shedding in LVAD patients with NSB compared to patients without bleeding complications by flow cytometry [6,16,17]. However, it is unknown whether the alteration in GP surface expression is only caused by shear stress-induced receptor shedding or if the intracellular expression pattern of glycoproteins is predisposed in patients with higher bleeding risk.

In the present study, we aimed to investigate the mRNA and protein expression of the GPIb-IX-V receptor complex in LVAD patients with and without bleeding complications and the possibility of using GPIb-IX-V as a predictive marker for bleeding risk stratification. We suggest that the loss of functional GPIb-IX-V complex could be involved in the pathophysiological mechanism of shear stress-induced coagulation disorder and NSB complications in patients with LVAD.

## 2. Results

### 2.1. Patient Characteristics

Study patients were predominantly male (bleeder group: 82%; non-bleeder group: 87%, Table 1) with a mean age of 59.8 ± 9.6 years. Comparative examination of demographic and clinical characteristics prior to LVAD implantation showed that bleeders and non-bleeders were comparable regarding age at LVAD implantation, gender, BMI, blood type, smoking history, alcohol abuse and the underlying etiology (*p* > 0.05). LVAD pump characteristics in terms of pump speed, pump flow and pump power were comparable between the groups. All patients were placed on an anticoagulation regimen with phenprocoumon and additional patient-specific dosing of acetylsalicylic acid (ASS) or clopidogrel after LVAD implantation. Patients of the non-bleeder group were more often anticoagulated with ASS than patients of the bleeder group (*p* = 0.04) (Table 1).

There were no significant differences in the occurrence of thromboembolic events prior to LVAD implantation and during the follow-up period. Twenty-seven patients experienced at least one postoperative NSB, and 41% of these patients had more than one bleeding event. The majority of NSB incidents after LVAD implantation were gastrointestinal (GI) bleeding events (44%), seven bleeding events resulted from epistaxis, hemothorax occurred in four patients, followed by dermal-related causes (11%) and intracranial bleeding events (7%) (Table 2).

The bleeder group was further divided into patients with NSB within the first 3 months (bleeder ≤ 3 mo., *n* = 19) and patients with NSB after 3 months (bleeder > 3 mo., *n* = 8). In patients of the bleeder group ≤ 3 mo., the first bleeding event occurred after 5 ± 4 weeks, whereas bleeder > 3 mo. bled after 72 ± 59 weeks.

Blood count analysis revealed that erythrocytes, hematocrit and hemoglobin content were reduced (*p* < 0.01) in bleeders compared to non-bleeders at the time of laboratory measurements (Table 3). Platelet count and leukocytes, international normalized ratio (INR), as well as the inflammatory marker C-reactive protein and lactate dehydrogenase, were comparable in patients of the bleeder and non-bleeder groups (Table 3).

**Figure 1:**
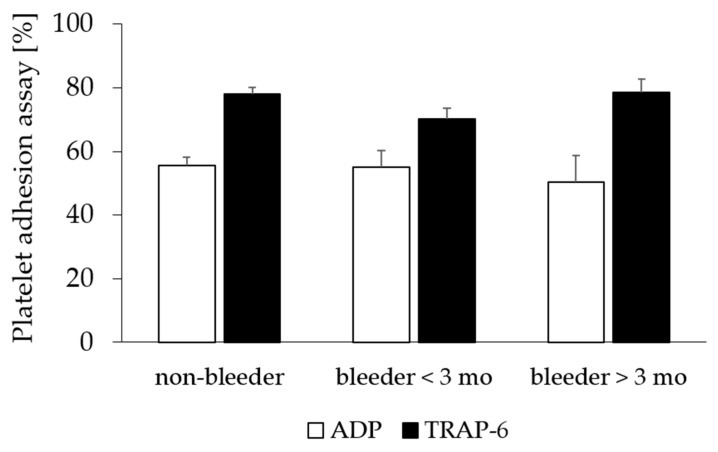
Platelet adhesion assay after LVAD implantation. Platelets aggregability of ADP and TRAP-6-stimulated platelets in patients without bleeding complications (non-bleeder) and patients experienced NSB within the first 3 months (bleeders < 3 mo) and more than 3 months after LVAD implantation (bleeders > 3 mo). All values are expressed as mean ± standard error. ADP, adenosine-diphosphate; NSB, non-surgical bleeding; TRAP-6, thrombin-receptor-activating peptide-6.

The comparison of postoperative platelet functional testing by platelet adhesion assay and vWF diagnostics revealed no significant difference between bleeders and non-bleeders in the study begin (*p* > 0.05) (Figure 1; Table 4).

### 2.2. Analysis of Platelet Receptor mRNA Expression

**Figure 2:**
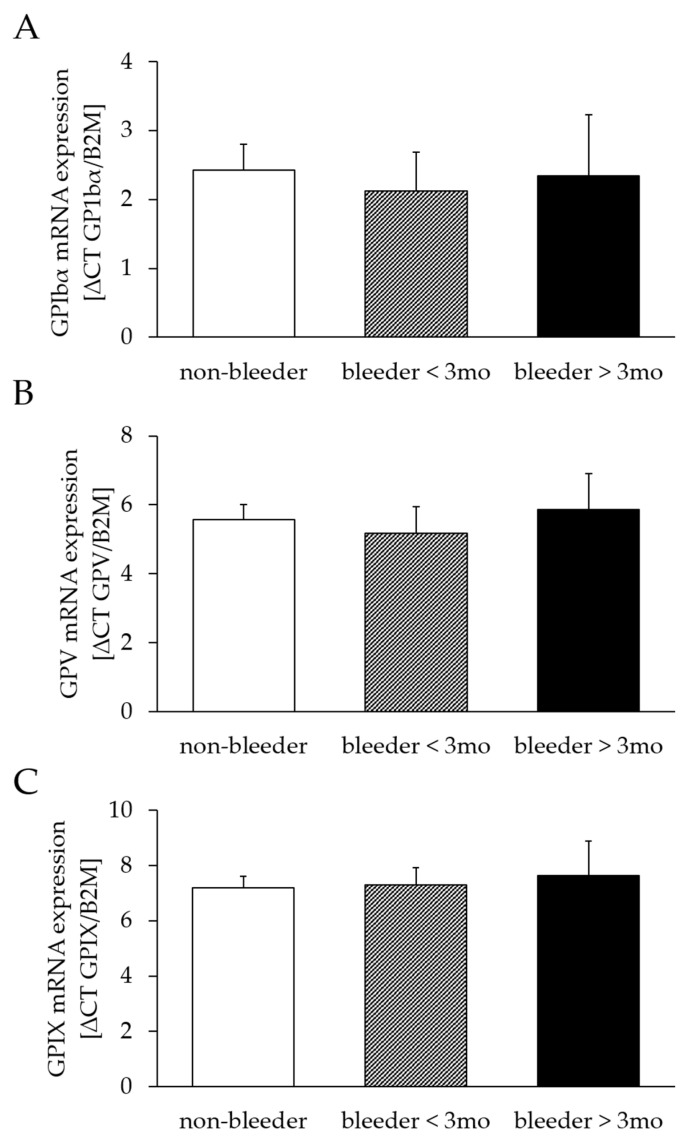
mRNA expression of GPIbα (**A**), GPV (**B**) and GPIX (**C**) after LVAD implantation. Relative mRNA expression was determined by real-time quantitative polymerase chain reaction, normalized to the housekeeping gene B2M and calculated by ∆CT. Study patients were retrospectively divided into three groups of non-bleeders and bleeders who experienced NSB within the first 3 months and more than 3 months after LVAD implantation. All values are expressed as mean ± standard error. B2M, beta-2 microglobulin protein; GP, glycoprotein.

The mRNA expression of GPIbα (bleeders: 2.19 ± 0.48; non-bleeders: 2.43 ± 0.39; *p* = 0.71), GPV (bleeders: 5.38 ± 0.63; non-bleeders: 5.56 ± 0.46; *p* = 0.82) and GPXI (bleeders: 7.41 ± 0.59; non-bleeders: 7.19 ± 0.44; *p* = 0.78) did not differ between bleeders and non-bleeders. A classification into early (first bleeding event within the first 3 months after LVAD implantation) and late bleeders (first bleeding event later than 3 months after LVAD implantation) confirmed the comparability of mRNA expression levels of GPIbα (bleeder ≤ 3 months: *p* = 0.67; bleeders > 3 months: *p* = 0.94), GPV (bleeder ≤ 3 months: *p* = 0.67; bleeders > 3 months: *p* = 0.81), and GPIX (bleeder ≤ 3 months: *p* = 0.89; bleeders > 3 months: *p* = 0.73) between bleeders and non-bleeders (Figure 2).

### 2.3. Analysis of Platelet Receptor Protein Expression

**Figure 3:**
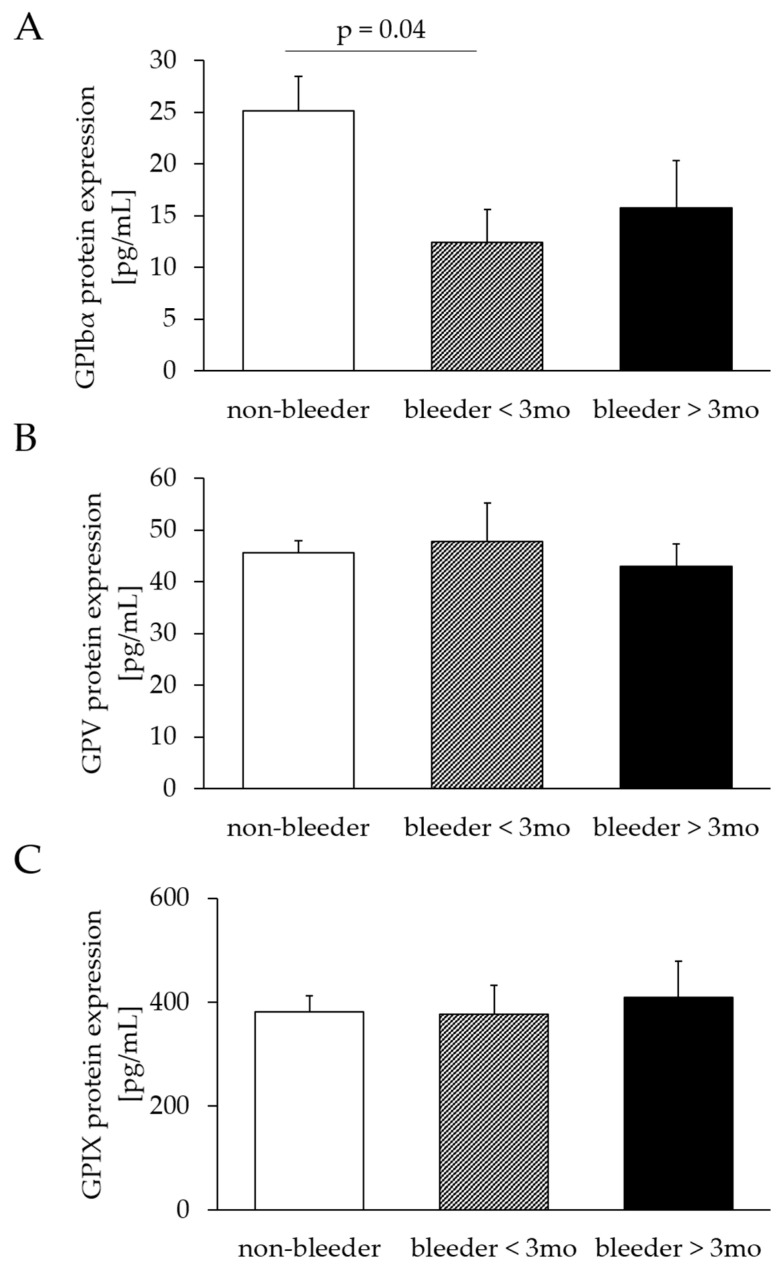
Comparison of GPIbα (**A**), GPV (**B**) and GPIX (**C**) protein expression after LVAD implantation. Protein expression was determined by enzyme-linked Immunosorbent assay and normalized to the ratio of total protein content to GPIbα, GPV or GPIX protein content. Study patients were retrospectively divided three groups of non-bleeders and bleeders who experienced NSB within the first 3 months and more than 3 months after LVAD implantation. All values are expressed as mean ± standard error. *p* ≤ 0.05 is considered statistically significant. GP, glycoprotein.

The protein expression of GPV (bleeders: 46.33 ± 5.47; non-bleeders: 45.53 ± 2.42; *p* = 0.88) and GPXI (bleeders: 386.50 ± 44.91; non-bleeders: 387.72 ± 32.87; *p* = 0.98) did not differ between bleeders and non-bleeders, whereas GPIbα protein expression was reduced (*p* = 0.02) in bleeders (bleeders: 16.46 ± 2.91; non-bleeders: 27.09 ± 3.52). The classification into early and late bleeders confirmed the comparability of protein expression levels of GPV (bleeder ≤ 3 months: *p* = 0.71; bleeders > 3 months: *p* = 0.70) and GPIX (bleeder ≤ 3 months: *p* = 0.95; bleeders > 3 months: *p* = 0.75) between bleeders and non-bleeders. The protein expression of GPIbα was decreased in bleeders ≤ 3 months (*p* = 0.04) but comparable in bleeders > 3 months (*p* = 0.31), compared to non-bleeders (Figure 3).

### 2.4. Assessment of Serum Levels of GPIbα

The mean values of plasma GPIbα were comparable in the bleeder group (57.43 ± 13.02 pg/mL) and in the non-bleeder group (53.96 ± 15.75 pg/mL; *p* = 0.33). The classification into early and late bleeders confirmed the comparability of protein expression levels of GPIbα (bleeder ≤ 3 months: *p* = 0.29; bleeders > 3 months: *p* = 0.87) between bleeders and non-bleeders.

## 3. Discussion

NSB remains a frequent and severe complication in LVAD patients [18]. The present study examined the GPIb-IX-V platelet receptor complex in HM 3 patients with and without postoperative bleeding complications to evaluate the potential impact of differential expression levels of GPIb-IX-V on bleeding risk stratification. We noticed that the mRNA expression levels of the complex subunits GPIbα, GPIX and GPV did not differ in HM 3 patients who developed NSB when compared to patients without bleeding complications. However, the protein expression of the main subunit GPIbα was significantly reduced in patients who experienced their first bleeding event within 3 months after LVAD implantation. The observed changes of platelet receptor GPIbα may influence platelet physiology by reduced platelet adhesion capacities, which may lead to an impaired hemostatic process and the elevated propensity of bleeding in HM 3 patients.

Platelets are small, anucleate cell fragments that originate from the cytoplasm of bone marrow-derived megakaryocytes and circulate in high numbers in the human bloodstream. During the formation of platelets, platelet-specific granules, proteins and mRNA fragments were provided by the megakaryocyte [19]. Mature platelets have a restricted but present protein synthesis machinery. Schubert et al., summarized the findings of several groups that reported de novo protein synthesis for proteins such as fibrinogen, GPIb, GPIIb, GPIIIa, tissue factor and other proteins by blood platelets [20]. Thus, we suggest that the unaffected mRNA expression level of GPIbα, GPIX and GPV in bleeders and non-bleeders seen in our study cohort might not be due to platelet formation by megakaryocytes.

The analysis of protein expression revealed a significantly reduced GPIbα level in patients with early bleeding events compared to patients without bleeding complications. This argues for the development of molecular differences in mature platelets of patients at higher bleeding risk in the short-term postoperative period.

The GPIb-IX-V complex is one of the most abundant adhesion receptors exclusively expressed on megakaryocytes and platelets. GPIb-IX-V is involved in several physiological and pathophysiological processes, such as the unique ability to mediate the initial steps of platelet adhesion to injured vessel cells and the recruitment of inflammatory monocytes [21,22]. GPIbα is the key domain of this receptor complex because it contains both the vWF and the thrombin binding site and is, therefore, critically involved in hemostasis and thrombus formation [7]. Compared to other receptor subunits, the large extracellular domain of GPIbα, with a mass of 135 kDa, is potentially more susceptible to structural damage under non-physiological conditions of high shear stress as in LVAD patients [23]. Given the functional importance of GPIbα interacting with vWF for platelet activation, the decreased intracellular protein expression of GPIbα observed in this study could be one possible explanation for a higher bleeding tendency within 3 months of LVAD implantation. This finding is in concordance with our previous study showing that the surface level of GPIbα is reduced in LVAD patients with NSB compared to patients without coagulation disorder by flow cytometry [16]. NPPS induces not only platelet activation but also the shedding of platelet receptors. Receptor shedding refers to the irreversible removal of surface receptors by proteolytic degradation and the release of soluble ectodomain fragments. Ectodomain shedding and proteolysis of GPIb-IX-V is regulated by a disintegrin and metalloproteinase (ADAM)-family members. ADAM17 cleaves GPIbα [24,25,26,27]. Besides direct mechanical damage, ADAM-induced receptor shedding can trigger intracellular signaling that may influence the GP expression level under pathophysiological conditions. The loss of platelet receptors could be a protective mechanism for downregulating hyperactive platelets during elevated shear stress, thereby reducing thrombus formation but simultaneously increasing bleeding tendency [28]. In this study cohort, the comparable plasma levels of GPIbα in bleeders and non-bleeders suggest that platelet receptor shedding may not be the only mechanism responsible for NSB.

Protein synthesis in platelets is limited, but posttranslational modifications (e.g., phosphorylation or glycosylation) of platelet adhesion receptors regulate the functional diversity of the receptors [19]. These modifications might affect GPIbα protein expression and platelet function early in pathological processes induced by non-physiological shear stress conditions in LVAD patients.

In a recent study, NSB in LVAD patients has been associated with genetic polymorphism of genes that are involved in coagulation [29]. Previously, Potapov and colleagues showed that VAD patients with genetic variation of platelet receptor GP IIb/IIIb developed more bleeding complications than patients with the alternative genotype [30]. Consequently, genetic polymorphism of platelet receptor GPIb-IX-V might contribute to the development of bleeding complications after LVAD implantation.

Platelets damaged by NPSS can be recognized and removed from circulation by apoptosis. On the one hand, GPIbα may act as one of the mechanoreceptors that transmit apoptotic signals inside the platelet [31]. On the other hand, Mondal et al. showed that the generation of endogenous reactive oxygen species (ROS) mediates the intrinsic pathway of platelet apoptosis [32]. Further, they suggest a possible role of oxidative stress in platelet receptor shedding leading to NSB in LVAD patients [6].

Platelet defects can also affect the normal expression and function of GPIbα that may result in bleeding complications. Animal studies demonstrated that genetic disruption of normal GPIbα impaired platelet adhesion capacity and attenuated the thrombotic propensity of platelets [33]. In humans, the Bernard-Soulier syndrome is characterized by the deficiency or dysregulation of the GPIb-IX-V complex, resulting in an increased bleeding propensity [22].

The concept of an automated pump speed adjustment in LVADs is a potential treatment option to reduce the number of complications, such as thrombosis, suction events or bleeding [34]. In this study, the analysis of pump characteristics revealed comparability between bleeders who experienced NSB within the first 3 months and more than 3 months after LVAD implantation. Additionally, the protein expression of GPIbα did not correlate with pump speed (r = 0.107), pump flow (r = 0.054) or pump power (r = 0.062).

Furthermore, the timing of the first bleeding event seemed to be an important factor. Shortly after LVAD implantation, nearly all patients develop the acquired von Willebrand syndrome (AVWS), indicated by the loss of large multimers of the vWF and reduced adhesive activity of blood platelets. The AVWS may influence bleeding episodes in LVAD patients, but not all patients develop post-implant bleeding complications [35,36]. In this study, the reported differences in GPIbα expression occurred within the first 3 months post-implantation but not in patients with NSB in the later follow-up. This is consistent with the higher number of patients with early (<3 months) bleeding events compared to patients with later bleeding events. Because GPIbα mediates platelet adhesion, the loss of this receptor argues for dysfunctional platelets in these patients and may increase the perioperative bleeding risk.

Anticoagulation is a major factor in preventing pump thrombosis but simultaneously increases the risk of bleeding complications [18]. All patients were regularly monitored for platelet adhesion and INR. In this study, the therapeutic values for clinical markers of clotting time, platelets aggregability and INR, as well as platelet counts, were comparable between the two groups. This suggests an optimal setting of anticoagulation treatment during the follow-up period and may not be causal for NSB complications. Additionally, we showed in our previous study that platelets aggregability did not differ between bleeders and non-bleeders in unstimulated, ADP- and TRAP-6-stimulated platelets [16]. Moreover, to our knowledge, there is no direct impact of administrated anticoagulation on the investigated receptor complex GPIb-IX-V.

Low hemoglobin, hematocrit and erythrocytes values were observed in both study cohorts independent of the occurrence of hemorrhagic events. This could be explained by the underlying chronic heart failure in LVAD patients, which may be associated with chronic anemia [37].

We acknowledge that there are some limitations in this monocentric observational study. First, drug-drug interactions or new side effects of anticoagulation medication on the altered expression level of the investigated GPIb-IX-V platelet receptor complex cannot be excluded. In addition, this study did not examine the differential effect of major and minor bleeding events on GPIb-IX-V because the observed type of bleeding complication was limited in each subgroup. In future studies, it would be useful to analyze the protein expression of GPIb-IX-V after subcellular fractioning of blood samples to distinguish between cytosolic and membranous parts of the receptor components in platelets. Moreover, the regulations of protein receptor expression through posttranslational modifications or the shear-induced metalloproteinase-dependent receptor shedding are potential targets of interest. A larger cohort of LVAD patients is needed to confirm our initial findings and improve predictability for clinical practice. It is important to verify whether the intracellular decreased expression level of GPIbα is a reliable predictor for bleeding complications after LVAD implantation. In patients with low GPIbα expression, individualized reduced anticoagulation is conceivable to avoid potential bleeding complications. Thereafter, the adjustment of patient-specific anticoagulation, lowering the target INR or modulation of pump characteristics could be considered. With increasing insight into the pathophysiological mechanism of NSB, a new target could be developed to attenuate bleeding complications in LVAD patients.

## 4. Materials and Methods

### 4.1. Study Groups and Clinical Characteristics

The study was conducted according to the Declaration of Helsinki, and the study protocol was approved by the local ethics committee of the Medical Faculty from the University of Leipzig, Germany (ID:225/17-ek). All patients gave their written informed consent prior to the study beginning. We studied 82 end-stage HF patients who underwent LVAD implantation between January 2016 and November 2020 at the Heart Center in Leipzig, Germany. All patients received the HM 3 LVAD pump. Study patients were retrospectively divided into two groups of patients with NSB (bleeder group: *n* = 27) and without bleeding complications (non-bleeder group: *n* = 55). NSB was defined as any sort of bleeding, including gastrointestinal bleeding (e.g., melena or upper gastrointestinal bleeding according to Forrest classification), hematothorax, hematoma, epistaxis or intracranial bleeding that occurred during their postoperative follow-up. Patients with a history of bleeding or anticoagulation disorder prior to LVAD implantation were excluded from our study. Patient characteristics and clinical data, including age at implantation, gender, BMI, etiology, smoking history, and alcohol abuse, were recorded. Additionally, we documented LVAD pump characteristics, medication of anticoagulation, differential blood count and vWF diagnostics. During the postoperative course, the occurrence of thromboembolic and bleeding events were gathered.

### 4.2. Sample Collection

Citrated whole blood and serum were obtained consecutively from LVAD patients at their regular follow-up visits. Sera were centrifuged at 2000× *g* for 10 min, aliquoted and frozen at −20 °C for analysis. Platelet-rich plasma (PRP) was prepared from whole blood by centrifugation at 200× *g* for 15 min. Afterward, PRPs were aliquoted and further processed by subsequent centrifugation at 1500× *g* for 20 min. After discarding the supernatant, the generated platelet pellet was snap-frozen and stored at −80 °C until further analysis.

### 4.3. RNA Extraction and cDNA Synthesis

Platelet RNA isolation was performed using TRIzol™ Reagent (Thermo Fisher Scientific, Waltham, MA, USA). Initially, the snap-frozen platelet pellets were incubated with 400 µL TRIzol at RT for 5 min to lyse cells. Then, 80 µL chloroform was added to the suspension and shaken by hand for 1 min. After an additional incubation step for 2–3 min, the sample was centrifuged at 12,000× *g* and 4 °C for 15 min. The upper aqueous RNA-containing phase was transferred into a new reaction tube, followed by RNA precipitation with 250 µL of 100% isopropanol and subsequent incubation for 10 min. The RNA-isopropanol mixture was centrifuged at 12,000× *g* and 4 °C for 10 min. After discarding the supernatant, the RNA pellet was washed with 250 µL of 75% ethanol and centrifuged again at 4 °C and 7500× *g* for 5 min. Finally, the dried RNA pellet was resuspended in 50 µL RNase-free water and incubated in a heat block at 55 °C for 15 min. The quantity and quality of RNA concentration were determined with the microplate reader Infinite™ 200 PRO and i-control™ software (both Tecan, Männedorf, Switzerland). The subsequent synthesis of cDNA was performed with the QuantiNova reverse transcription kit in 20 µL reaction volumes according to the manufacturer’s instructions (Qiagen GmbH, Hilden, Germany). Primer sequences used for the amplification of cDNA in a real-time quantitative polymerase chain reaction (RT-qPCR) were purchased from TIB Molbiol (TIB Molbiol synthesis laboratory GmbH, Berlin, Germany) (Table 5).

### 4.4. Real-Time Quantitative PCR Analysis

RT-qPCR reactions were conducted in 20 µL volumes in 96-well RT-qPCR plates (both, Genaxxon bioscience GmbH, Ulm, Germany). Reaction mixes were prepared by adding QuantiNova SYBR Green PCR Master Mix, oligonucleotide forward and reverse primers, as well as template cDNA following the manufacturer’s specifications of the QuantiNova SYBR Green PCR Kit (Qiagen). After the initial heat activation of the samples at 95 °C for 2 min, the cycling program consists of two repeating (40×) steps denaturation at 95 °C for 10 s and combined annealing and extension step at 60 °C for 20 s. The emerging fluorescence signal was selected using the LightCycler 480 (Roche Diagnostics, Mannheim, Germany). Melting curve analysis was performed to verify the specificity of RT-qPCR products.

### 4.5. Protein Extraction and Quantification

Snap-frozen platelet pellets were homogenized in lysis relaxing buffer (126 mM KCl, 90 mM HEPES, 50 mM EGTA, 36 mM NaCl, 10 mM Creatininphosphate, 8 mM ATP, 1 mM MgCl_2_) and 1 × protease and phosphatase inhibitor cocktail (Thermo Fisher Scientific), and sonicated twice in ice water for 5 min, with a 5 min break in between. Afterward, protein content was quantified using the Pierce™ Microplate BCA protein assay Kit (Thermo Fisher Scientific).

### 4.6. Elisa

Protein concentrations of GPIbα, GPV and GPIX were determined using the human platelet glycoprotein Ib alpha chain ELISA Kit and the human platelet glycoprotein IX ELISA Kit (Signalway Antibody LCC, College Park, MD, USA) and the Fine Test^®^ ELISA Kits for GPV (Whuan Fine Biotech Co., Ltd., Wuhan, China). Additionally, serum levels of GPIbα were assessed using the human platelet glycoprotein Ib alpha chain ELISA Kit. The assays were performed according to the manufacturer’s instructions. Measurements were recorded at 450 nm with the microplate reader Infinite™ 200 PRO and i-control™ software (both Tecan, Männedorf, Switzerland).

### 4.7. Statistical Analysis

Statistical analyses were performed using Intel SPSS statistical software version 28 (IBM Corp., New York, NY, USA, 1989). Data are presented as mean ± standard deviation or as percentage proportion. Group comparisons of categorical data were performed using Pearson’s Chi-Squared test, Fisher’s exact test or the Yates continuity correction. Unpaired t-tests were used in the case of two-group comparisons for metric parameters. The statistical significance level was assigned at *p* ≤ 0.05 (two-sided).

## 5. Conclusions

In summary, our study documented changes in the protein expression of GPIbα in patients who experienced NSB complications within the first 3 months of HM 3 implantation compared to patients without NSB. These changes may contribute to dysfunctional platelets and therefore play a role in the pathophysiology of NSB and help understand the increased risk of bleeding in patients with LVAD.

## Figures and Tables

**Table 1:** Demographic and clinical characteristics of bleeders and the non-bleeders prior to LVAD implantation and LVAD-related parameters.

Parameter	Bleeders (*n* = 27)	Non-Bleeders (*n* = 55)	*p*-Value
age at LVAD implantation [y]	60.9 ± 9.9	59.2 ± 9.5	0.46
male gender	22 (82%)	48 (87%)	0.52
body mass index [kg/m^2^]	28.3 ± 5.5	28.6 ± 5.5	0.86
blood type *			0.81
A	11 (41%)	28 (52%)	
B	4 (15%)	5 (9%)	
AB	1 (4%)	2 (4%)	
0	11 (41%)	19 (35%)	
etiology of heart disease			0.35
NICM	12 (44%)	32 (58%)	
ICM	15 (56%)	23 (42%)	
alcohol abusus			0.62
current	5 (19%)	6 (11%)	
former	14 (52%)	25 (45%)	
occasional	5 (19%)	13 (24%)	
unknown	3 (11%)	11 (20%)	
smoking			0.61
current	3 (11%)	10 (18%)	
former	16 (59%)	29 (53%)	
never	3 (11%)	9 (16%)	
unknown	5 (19%)	7 (13%)	
pump characteristics			
pump speed [rpm]	5141 ± 571	5271 ± 2665	0.16
pump flow [L/min]	4.2 ± 0.6	4.3 ± 0.7	0.27
pump power [W]	3.9 ± 0.5	4.0 ± 0.4	0.45
pulse index	4.5 ± 2.0	4.4 ± 1.6	0.82
anticoagulation at study begin			
Phenprocoumon/warfarin	27 (100%)	55 (100%)	1.00
clopidogrel	7 (26%)	11 (20%)	0.58
acetylsalicylic acid	16 (59%)	45 (82%)	0.04
prasugrel	1 (4%)	0 (0%)	0.33

* one patient is unknown. ICM, ischemic cardiomyopathy; LVAD, left ventricular assist device; NICM, non-ischemic cardiomyopathy.

**Table 2:** Thromboembolic and hemorrhagic events after LVAD implantation.

Parameter	Bleeders (*n* = 27)	Non-Bleeders (*n* = 55)	*p*-Value
thromboembolic event prior LVAD implantation			
ischemic stroke	3 (%)	9 (%)	0.74
pulmonary embolism	1 (%)	1 (%)	1.00
venous thrombosis	2 (%)	5 (%)	1.00
ventricle thrombosis	3 (%)	14 (%)	0.16
myocardial infarction	7 (%)	15 (%)	1.00
hemorrhagic events after LVAD implantation *			
gastrointestinal tract bleeding	12 (44%)	-	
vascular (epistaxis)	7 (26%)	-	
haemothorax	4 (15%)	-	-
dermal bleeding (haematoma)	3 (11%)	-	
intracranial bleeding	2 (7%)	-	
other	4 (15%)	-	
thromboembolic event after LVAD implantation			
ischemic stroke	0 (0%)	3 (6%)	1.00
pAVK	2 (7%)	3 (6%)	1.00
pump thrombosis	0 (0%)	1 (2%)	1.00
rethoracothomy after LVAD implantation	13 (48%)	29 (53%)	0.82

* including multiple bleeding events; LVAD, left ventricular assist device.

**Table 3:** Differential blood count parameters of bleeders and non-bleeders after LVAD implantation at the time-point of laboratory measurements.

Parameter	Bleeders (*n* = 27)	Non-Bleeders (*n* = 55)	*p*-Value
erythrocytes [Tpt/L]	3.8 ± 0.8	4.4 ± 0.8	<0.01
hematocrit	0.3 ± 0.1	0.4 ± 0.1	<0.01
hemoglobin [mmol/L]	7.2 ± 1.3	8.3 ± 1.5	<0.01
platelets [×109/L] at study begin	211 ± 82	221 ± 67	0.55
platelets [×109/L] at bleeding event	234 ± 115	-	-
leucocytes [Gpt/L]	7.7 ± 2.3	7.8 ± 1.9	0.83
INR	2.1 ± 0.5	2.2 ± 0.4	0.10
CRP [mg/L]	12.4 ± 16.4	8.2 ± 10.3	0.23
LDH [µmol/(L/s)]	3.9 ± 1.0	4.2 ± 0.8	0.28

CRP, C-reactive protein; INR, international normalized ratio; LDH, lactate dehydrogenase.

**Table 4:** Platelet adhesion assay, von Willebrand and Factor VIII diagnostics in bleeders and non-bleeders after LVAD implantation at the time-point of laboratory measurements.

Parameter	Bleeders (*n* = 27)	Non-Bleeders (*n* = 55)	*p*-Value
Platelet adhesion assay [%]			
ADP	54 ± 24	56 ± 20	0.70
TRAP-6	73 ± 15	78 ± 15	0.13
VASP-phosphorylation	70 ± 22	77 ± 18	0.12
aPTT [s]	44.2 ± 7.4	42.6 ± 6.4	0.32
vWF antigen [%]	198.6 ± 65.5	176.1 ± 61.0	0.26
vWF activity [%]	136.7 ± 28.7	128.5 ± 34.1	0.43
vWF CB activity [%]	135.5 ± 41.9	116.1 ± 37.3	0.22
factor VIII, procoagulant [%]	188.5 ± 62.4	171.0 ± 57.5	0.36

aPTT, activated partial thromboplastin time; ADP, adenosine diphosphate; CB, collagen-binding; TRAP-6, thrombin receptor-activating peptide; VASP, vasodilator-stimulated phosphoprotein; vWF, von Willebrand factor.

**Table 5:** Primer sequences for RT-qPCR.

Target	Forward Primer 5′-3′	Reverse Primer 5′-3′
GPIbα	TGCCAGATCTCACGGTGAAC	AACGAGTGCTCACATCCTGAT
GPV	CCACTTGCTTTACATCCATGCA	GGAAGAACAAATGAGACTGTGACA
GPIX	AACAACAGCCTTCAGTCCGT	CAGAGGCGCAGATAGGTGAG
B2M	AGTATGCCTGCCGTGTGAAC	GCAAGCAAGCAGAATTTGGA

B2M, beta-2 mircroglobulin; GP, glycoprotein; RT-qPCR, real-time quantitative polymerase chain reaction.

## Data Availability

Not applicable.
